# Sympathetic Nervous System and Exercise Affects Cognition in Youth (SNEACY): study protocol for a randomized crossover trial

**DOI:** 10.1186/s13063-021-05096-w

**Published:** 2021-02-18

**Authors:** Lauren B. Raine, Katherine McDonald, Tatsuya T. Shigeta, Shu-Shih Hsieh, Jennifer Hunt, Nathan A. Chiarlitti, Michelle Lim, Kristen Gebhardt, Nina Collins, Michael De Lisio, Sean P. Mullen, Arthur F. Kramer, Charles Hillman

**Affiliations:** 1 Department of Psychology, Northeastern University, 635 ISEC, 360 Huntington Ave, Boston, MA 02115 USA; 2 School of Human Kinetics and Department of Cellular and Molecular Medicine, University of Ottawa, Ottawa, Canada; 3 Tufts University, Medford, USA; 4 Department of Kinesiology and Community Health, University of Illinois at Urbana-Champaign, Champaign, USA; 5 Beckman Institute, University of Illinois at Urbana-Champaign, Champaign, USA; 6 Department of Physical Therapy, Movement, & Rehabilitation Sciences, Northeastern University, Boston, USA

**Keywords:** Brain function, Executive control, Neuroimaging, Physical activity, Children

## Abstract

**Background:**

There is an increasing prevalence of physical inactivity during childhood, which is associated with a variety of health problems. However, the mechanisms by which acute exercise benefits cognition in childhood remains unknown. Here we describe the protocol for a randomized crossover trial called SNEACY (*Sympathetic Nervous System & Exercise Affects Cognition in Youth*), a study designed to better understand mechanisms linking acute exercise and cognition in 9–10-year-old healthy, cognitively normal children.

**Methods:**

Children from the Greater Boston, MA region will be recruited to participate in this single center study. A randomized crossover design will be utilized, such that participants will act as their own controls, through initial randomization to condition assignment and condition counterbalancing across participants. One hundred three children will participate in three randomized acute interventions: moderate intensity treadmill exercise (20 min, 70–75% of their maximal heart rate), seated rest (20 min), and a Trier Social Stress Test for Children (14 min). These visits will occur on 3 three separate days, approximately 5–8 days apart. Before and after each intervention, children complete a variety of cognitive tasks measuring attentional inhibition while their neuroelectric activity is recorded. Variables of interest include EEG data, accuracy and reaction time, academic achievement, and salivary alpha amylase. Academic achievement is also assessed following interventions. In addition, children provide passive drool samples throughout the interventions to measure various biomarkers that may explain the acute exercise benefit on cognition.

**Discussion:**

The results from this study could influence educational and public health recommendations to enhance cognition and learning in pre-adolescent children.

**Trial registration:**

ClinicalTrials.gov NCT03592238. Registered on 19 July 2018

## Background

Despite widespread health campaigns, children have become increasingly inactive and unfit [1], with > 50% of children not meeting the daily recommended 60 min of moderate-to-vigorous physical activity (MVPA) [2]. Adverse effects of inactivity during childhood have been observed for their physical health [3], as well as their psychosocial well-being [4]. Inactivity during childhood tracks into adolescence and adulthood, resulting in earlier mortality and greater morbidity associated with chronic diseases including cardiovascular disease and type 2 diabetes. However, largely absent from public health concerns is the effect of physical inactivity on brain health and cognition. In contrast, there is a growing literature detailing the beneficial effects of physical activity (PA) on brain health, cognition, and academic performance [5–7].

In particular, growing evidence indicates that an acute bout of PA has beneficial effects on brain function, cognition, and scholastic achievement [8–10]. For example, 20 min of moderate intensity walking improves neural and behavioral correlates of inhibition and academic performance [8–10]. That is, following 20 min of walking, reports indicated that children exhibit enhanced brain function, improved cognitive performance during tasks demanding greater amount of inhibition, and higher academic achievement in mathematics and reading compared to 20 min of rest [9]. The technique used to measure brain function was event-related brain potentials (ERPs), a non-invasive measure of neuroelectric activity during task engagement and response selection. One ERP component, known as the P3, is related to the allocation of attentional resources [11, 12] and the speed of stimulus classification during stimulus engagement [13, 14].

However, minimal evidence exists regarding potential *mechanisms* underlying the transient effects of acute PA on brain and cognition. One potential mechanism involves phasic shifts in the sympathetic nervous system (SNS), as measured via the biomarker: salivary alpha-amylase (sAA). sAA is an enzyme produced by the salivary glands, which is modulated by physical and psychosocial stress as part of the first wave of the “fight or flight” response [15]. The second wave of the stress response involves the release of the glucocorticoid, cortisol from the adrenal cortex. sAA and cortisol have also been related to alterations in executive control functions (i.e., inhibition, working memory, cognitive flexibility) and academic achievement in children [16].

The SNEACY study was designed to address possible mechanisms by which acute PA acts on cognition and brain function (as measured by ERPs) in typically developing, healthy children. This lack of understanding constitutes a significant gap in the research, which is reflected in a rapid decline of PA opportunities for children throughout the school day. Thus, this study will examine the extent to which PA influences the cognitive and brain health of children. Results are expected to be particularly relevant to public health and education with potential implications for future policies aimed at improving physical and mental health during childhood. In particular, the primary goal of the SNEACY study is to discover the role of SNS activity as a mediator of the effects of a single bout of PA on brain function, cognition, and academic achievement in preadolescent children.

## Material and design

### Summary of aims

**Fig. 1:**
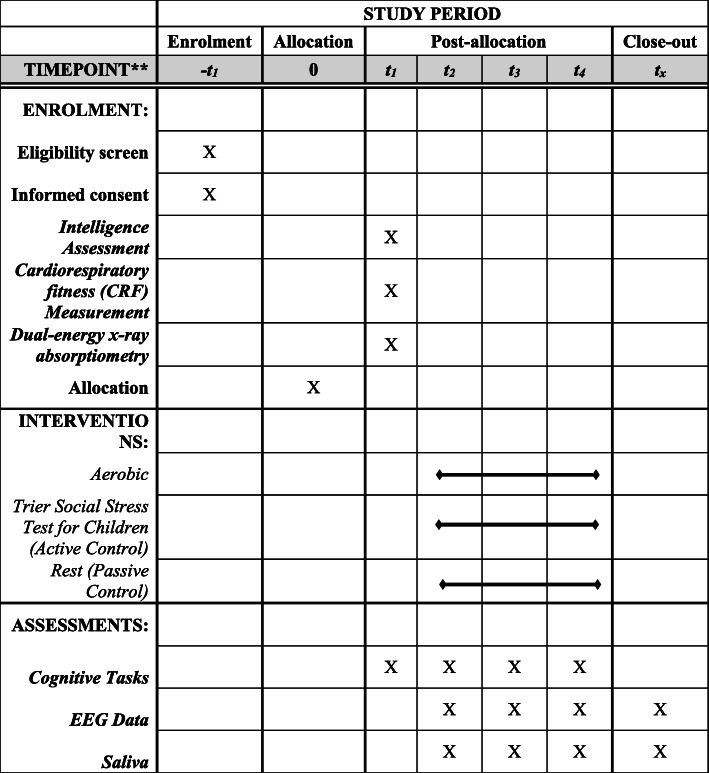
SNEACY schedule of enrollment, interventions, and assessments

The objective of the SNEACY study is to test several aims: (1) Investigate basic laboratory and applied scholastic performance aspects of brain function, cognition, and academic achievement following aerobic exercise compared to active (i.e., Trier Social Stress Test for Children (TSST); a commonly used psychosocial stressor) and passive (i.e., seated rest) control conditions; and (2) Investigate phasic changes in SNS activation (as indicated by sAA) as a potential mechanism for the transient beneficial effect of exercise on brain function, cognition, and academic achievement. Phasic activation refers to brief, rapid changes in the SNS [17] in response to salient or task-relevant information in the stimulus environment [18]. Phasic sAA has been shown to be related with attention, memory, and various academic outcomes [16, 18–22]. SNEACY is a randomized crossover trial with three acute interventions: 20 min of moderate intensity treadmill exercise, 20 min of seated rest, and a 14-min TSST for Children. The TSST and seated rest interventions serve as active and passive control conditions, respectively, for the experimental intervention involving moderate intensity exercise. As a result of this design, changes observed in the aerobic exercise intervention can be attributed to the exercise component of the intervention rather than to other, potentially confounding factors, such as expectancy effects, stress, or elevated heart rate. The study will enroll and randomize 103 typically developing, 9–10-year-old children. We hypothesize that exercise-induced phasic increases in SNS activity, indicated by sAA levels, will mediate the effect of a single bout of exercise on brain function (i.e., P3 amplitude and latency), cognition (i.e., executive control), and standardized achievement test performance (i.e., mathematics, reading). To further explore a relation between exercise-induced change in sAA with brain and cognition, baseline and stress-induced (i.e., exercise, TSST) change in cortisol will be investigated as a potential factor in the analytical model. All participants provide written assent/consent (and legal guardians provide written informed consent) by a trained research technician in accordance with the Institutional Review Board of Northeastern University. The study was designed in accordance with the SPIRIT statement, details are provided in Fig. 1.

### Primary outcome

The primary outcomes are changes in cognitive performance following each of the three acute interventions. Specifically, we predict that exercise-induced phasic increases in SNS activity, indicated by sAA levels, will mediate the effect of a single bout of exercise on brain function (i.e., P3 amplitude and latency), cognition (i.e., executive control), and standardized achievement test performance (i.e., mathematics, reading).

### Recruitment

Recruitment for SNEACY began in February of 2019, and the goal is to enroll 103 (60 female) children by December 2021. Participants are typically developing, healthy 9–10-year-old children from the Greater Boston, MA region. The goal for recruitment of racial and ethnic minorities is in proportion to the demographic representation in Boston, MA (77.4% Caucasian, 13.2% African American, 17.4% Latino/Hispanic, 5.4% Asian, 1.2% Native American, 0.2% Native Hawaiian and Other Pacific Islander, and 2.5% multiracial). Participant recruitment occurs through local media outlets, community websites, parent group website, University list-serves, contact with local youth organizations, and fliers. To recruit racial and ethnic minorities, diverse neighborhoods in the greater Boston area will be a focus of recruitment, particularly in terms of community centers, libraries, and local newspapers. Fliers contain a general introduction of the research and the contact information of the research team so that interested individuals initiate contact with the research team directly. Recruitment, enrollment, and randomization occur continually.

### Eligibility

The eligibility criteria were designed to recruit children who are typically developing, generally healthy, and able to safely exercise. These criteria were developed to ensure participant safety, as well as a maximal generalization to children broadly (see Table 1).

**Table 1:** Inclusion-exclusion criteria for SNEACY participants

Inclusion	Exclusion
1. Parental/guardian consent and participant assent.	Non-consent of guardian or non-assent of participant.
2. 9–10 years of age.	Above/below 9–10 years of age.
3. Capable of performing exercise based on PAR-Q.	Any physical disability that prohibits exercise.
4. Normal/average IQ or above (i.e., > 85).	IQ < 85.
5. Participants must have not yet reached, or be in the earliest stages, of puberty, as measured by a modified test of the Tanner Staging System (Tanner score < 2).	Tanner score > 2.
6. No prior diagnosis of cognitive or physical disability, including ADHD, uncontrolled asthma, epilepsy, chronic kidney disease, and dependence upon a wheelchair/walking aid.	Prior diagnosis of cognitive or physical disability, including ADHD, uncontrolled asthma, epilepsy, chronic kidney disease, and dependence upon a wheelchair/walking aid.
7. Free of any type of anti-psychotic, anti-depressant, anti-anxiety medication as well as medications used for ADD/ADHD (use of any anti-psychotic, anti-depressant, anti-anxiety, and ADD/ADHD medications).	Use of any type of anti-psychotic, anti-depressant, anti-anxiety medication as well as medications used for ADD/ADHD (use of any anti-psychotic, anti-depressant, anti-anxiety, and ADD/ADHD medications).
8. Normal or corrected-to-normal vision based on the minimal 20/20 standard in order to complete the cognitive task (below 20/20 vision).	Normal or corrected-to-normal vision below 20/20 vision.
9. Able to speak and read English.	Not fluent in English.

### Screening

Questions about a child’s age, individualized education plan (IEP), medical history, safety to complete exercise (PAR-Q), and ADHD status are asked in an initial phone screen. Only children who meet these criteria remain eligible and are scheduled for the first day of testing. On the first day of testing, children are screened for IQ scores and Tanner status.

### Day 1 procedure

At the first visit, participants and their legal guardians complete a detailed health and demographic questionnaire, pre-participation health screening questionnaire, the ADHD Rating Scale IV, the Pittsburg Sleep Quality Index questionnaire, and the modified Tanner pubertal self-assessment questionnaire [23]. Parents/guardians are given the option of taking home the pubertal self-assessment questionnaire in case they prefer that their spouse complete the form or if they would rather respond to it at home. A trained experimenter administers the Edinburgh Handedness Inventory to the child. The child is then administered the KBIT-2 [24] to measure their intelligence quotient (IQ). All tests are entered into a database by staff, from which standardized scores and percentiles are created. Participants who meet all inclusion criteria are invited to participate in three additional days of testing. The first session lasts approximately 2.5 h.

#### Intelligence assessment

Children complete the Kaufman Brief Intelligence Test-2 (KBIT-2) [24] to assess intelligence. The KBIT-2 is a commercially available paper and pencil-based assessment of cognitive abilities that has been age normed.

#### Cardiorespiratory fitness (CRF) measurement

Children complete a VO_2_max test on a motorized treadmill (Treadmill: Trackmaster TMX428; Metabolic cart: COSMED Quark CPET OMNIA, Concord, CA) using a modified Balke protocol at the end of their first session. This test is conducted based on recommendations from the American College of Sports Medicine. Three trained, first-aid and CPR-certified, experimenters will conduct the exercise test. Participants complete a brief warmup and then run at a constant speed with incremental intensity increases through grade inclines of 2.5% every 2 min until volitional fatigue. The speed is determined based on the child’s height, running stride, and overall ability of the child. Participants wear a heart rate (HR) monitor during the test to determine maximal heart rate. Ratings of perceived exertion (RPE) are assessed every 2 min using the children’s OMNI scale [25]. Relative peak oxygen consumption is expressed in ml/kg/min and based upon maximal effort as evidenced by (1) a plateau in oxygen uptake corresponding to an increase of less than 2 ml/kg/min despite an increase in exercise workload, (2) a peak HR ≥ 185 bpm [26, 27] and a HR plateau [26], (3) RER ≥ 1. 0 [28], and (4) ratings on the children’s OMNI scale of perceived exertion ≥8 [25]. Age- and gender-matched percentile values will be derived by using normative values [29]. In order to reduce collinearity between whole body adiposity and aerobic fitness, fat-free VO_2_max (ml/kg lean/min) will be calculated using an individual’s absolute VO_2_max and lean mass. This measure has greater validity than when comparing aerobic fitness in children of different body sizes [30].

#### Dual-energy X-ray absorptiometry

Previous research suggests that body composition can influence cognition and brain function and may influence the mechanisms by which cognitive changes occur following acute exercise. Thus, we collect body composition measures to determine if they serve as mediator variables. Height and weight are measured and body mass index (BMI) is calculated by dividing body mass (kg) by height (m) squared. Dual-energy X-ray absorptiometry (DXA) (GE Lunar iDXA, Madison, Wisconsin) measures bone mineral content, density, and whole-body and regional soft tissue composition. Participants are instructed to wear or change into shorts and a T-shirt containing no metal. Participants lie on their back on the DXA table for about 10 min, remaining motionless with their eyes closed. The DXA can separate body mass into fat and lean measures, allowing for the evaluation of fat mass without the confounding influence of other tissues.

#### Psychosocial assessments

##### ChEAT

The ChEAT-26 is a 26-item, self-report questionnaire that measures the level of atypical and disordered eating attitudes and behaviors. Respondents answer each of the EAT items using a 6-point Likert-type scale and indicate how frequently (1 = never; 6 = always) they experience various thoughts, feelings, or behaviors in relation to eating.

##### Children’s Depression Inventory 2 (CDI 2)-Short Version

The CDI is used to screen for depressive symptoms. The CDI is widely used to assess depressive symptomatology in children and adolescents aged 8–17 years. The scale is composed of 12 Likert-type items scored from 0 to 2, with higher scores reflecting more important depressive symptomatology.

##### State and Trait Anxiety Inventory for Children (STAIC)

The STAIC is made up of 2 scales of 20 items each, describing psychological manifestations of anxiety; the State scale describes the current state of anxiety, and the Trait scale describes a general propensity to react with anxiety to stressful events. Each item is scored from 1 to 3, the total score for each scale thus ranging from 20 to 60.

#### Days 2–4 procedure

At intervention visits (days 2, 3, and 4), the participant engages in the same protocol each day, with the sole exception of a different intervention each day. At the start of each visit, children are outfitted with a heart rate monitor to measure the intensity of the intervention. Upon entering the laboratory, children are asked to sit quietly for 10 min to allow their heart rate to return to baseline levels, after which they provide the first saliva sample by drooling into a tube. Participants are then fitted with an EEG cap. Next, they complete cognitive tasks on a computer while EEG data is simultaneously collected. Then, the child completes one intervention at each visit: (1) aerobic exercise, (2) quiet resting, or (3) the TSST. Upon completion of the intervention, participants provide another saliva sample. Participants then complete the same cognitive tasks again while EEG data is simultaneously collected. Participants will provide another saliva sample, and then they will complete an academic achievement test. At the end of the visit, the final saliva sample is collected. Visits 2–4 last approximately 2.5 h each. At the conclusion of study participation, no further ancillary or post-trial care was offered.

#### Interventions

Children are asked not to exercise or participate in structured physical activity prior to attending any of their sessions, and this is verified via self-report before each intervention session. Maximal heart rate (HRMax) is determined from the maximal exercise test completed on day 1 and is used to determine relative exercise intensity for days 2, 3, or 4. The interventions will be completed in a randomized crossover design, such that participants will act as their own controls, through initial randomization to condition assignment and condition counterbalancing across participants. With 3 interventions, there are a total of 6 different interventions (for example, aerobic-TSST-rest, aerobic-rest-TSST, TSST-rest-aerobic, TSST-aerobic-rest, rest-aerobic-TSST, rest-TSST-aerobic). A random number generator will be used to determine which intervention order a participant receives. See Table 2 for participant timeline.

**Table 2:** Participant timeline

Time schedule	Activity
6–30 months	Participants Enrolled
Approximately 1 week prior to intervention 1	Day 1
	**Days 2–4 protocol**
0–10 min	10-min rest/baseline
10–25 min	EEG cap preparation
25–30 min	Baseline saliva sample
30–45 min	Pre-intervention cognitive tasks
	Interventions
45–60 min	*Trier*
45–70 min	*Aerobic and rest*
70–75 min	Post-intervention saliva sample
75–90 min	Post-intervention cognitive tasks
90–95 min	Post-cognitive task saliva sample
95–115 min	Academic achievement testing
115–120 min	Intervention completion saliva sample

##### Aerobic

To assess the effect of a single bout of aerobic exercise on executive control, we use an exercise stimulus based on the American College of Sports Medicine guidelines for improving aerobic capacity, which consists of 21 min of exercise on a treadmill at 70–75% of the individual’s maximum heart rate. Participants engage in a 1-min warmup and a 1-min cooldown, with the majority of time (i.e., 19 min) spent exercising at 70–75% of HRmax. Participants’ exercise on a motor-driven treadmill at a constant speed during the 19-min period. Based on fitness level and participant ability, they may walk or run. Once the desired speed is set, the incline is adjusted to reach the desired intensity.

##### Trier Social Stress Test for children (active control)

Children participate in a modified laboratory stress protocol based on the TSST for children [31]. The TSST for children consists of a speech task in which children talk about themselves and complete a mental arithmetic task in front of a camera (which does not record the session, but is there simply to induce stress) and three neutral observers. Specifically, participants are asked to imagine that they are in a new class with 20 other students, and that their teacher has asked them to stand in front of the class and introduce themselves. Children are instructed to speak for 6 min about themselves, their personality, and why they believe that they would be liked by their classmates. In addition, they are asked to discuss at least one good and one bad aspect of themselves. The mental arithmetic task entails asking children to serially subtract the number 5 from a larger number as quickly as possible for 4 min. The TSST for children lasts approximately 14 min. Although the TSST for children is shorter in duration than the exercise task, prior research has shown that it successfully elevates sAA and CORT levels [32].

##### Rest (passive control)

Participants sit in a comfortable chair, placed in the same room as the motor-driven treadmill, for 20 min. Children are asked to sit quietly or read a book of their choosing (excluding schoolwork). They are instructed to minimize conversation.

#### Cognitive tasks

##### Go-NoGo task

In the Go condition, participants are asked to respond to an infrequent target stimulus (cartoon drawing of a lion), occurring on 20% of the trials, and not to respond to the frequent stimuli (cartoon drawing of a tiger), occurring on 80% of the trials. Next, participants complete the NoGo condition, wherein the rules are reversed. Participants respond to the frequent non-target stimuli, while not responding to the rare, target stimuli. This fixed task order is used to build a prepotent response to respond to the rare stimuli. Participants complete one block of 250 trials for each condition (Go, NoGo). Stimuli appear for 200 ms and a black background with a variables inter-stimulus interval (ISI) of about 1500 ms.

##### Flanker task

A modified flanker task consists of five 3-cm-tall white arrows focally presented on a black background of a computer screen. Participants are instructed to respond using a response pad as quickly and accurately as possible with a thumb press according to the directionality of the centrally presented target arrow amid either congruent (pointing in the same direction) or incongruent (pointing in the opposite direction) flanking arrows. Congruency and directionality are equiprobable. Participants complete one block of 156 trials, with stimuli presented for 150 ms with a variable ISI of (1300, 1500, and 1700 ms).

#### Neuroelectric assessment: electroencephalogram (EEG)

One of the primary aims of the SNEACY study is to examine the acute changes that occur in brain function following exercise intervention. Accordingly, on sessions 2, 3, and 4, children are fit with an EEG cap in order to measure brain activity before and after intervention. EEG activity is measured from 64 electrodes and 4 integrated bipolar leads for vertical and horizontal EOG (VEOG, HEOG), arranged according to the international 10–10 system using a Neuroscan Quik-cap (Compumedics, Inc., Charlotte, NC). EEG data is collected while cognitive tasks are completed.

#### Academic achievement testing

##### Wide Range Achievement Test 4 (WRAT4)

Participants complete the WRAT-4 assessment tests for reading (the number of words pronounced correctly) and mathematics (the number of mathematical computations completed correctly). The WRAT-4 is a commercially available paper and pencil based academic achievement assessment that has been age normed. The WRAT allows for repeated administration through the use of two equivalent forms. Administration of the WRAT-4 is conducted by trained experimenters with the duration of the assessment lasting ~ 30 min. Each child completes one version of the WRAT-4 following their rest session, and one version of the WRAT-4 following either their aerobic or TSST session, since only two versions of the task exist.

#### Saliva collection (sAA and cortisol)

Salivary samples for evaluation of sAA and cortisol are collected throughout the experimental manipulation at 4 timepoints. These collection timepoints include: before cognitive testing begins/baseline, following the intervention, following post-intervention cognitive tasks, and at the completion of the visit. At each collection time, participants are seated comfortably, leaning forward with arms crossed in front of them. Unstimulated salivary samples (at least 2 mL) are collected by the passive drool technique over a 2-min period. Samples are collected into pre-weighed saliva collection tubes and immediately placed on ice. After weighing collection tubes for quantitation of saliva volume and determination of flow rate ([post-collection tube weight − pre-collection tube weight]/2 min), samples are aliquoted and frozen at − 80 °C until analysis. sAA and cortisol are packaged, shipped on dry ice, and analyzed at the University of Ottawa. sAA and cortisol are measured using commercially available kits (α-amylase Saliva Enzymatic Assay and Cortisol Saliva ELISA, IBL International, Hamburg, Germany) according to manufacturer’s instructions. Briefly, on the day of analysis, saliva samples are thawed, centrifuged at 1500×*g* for 15 min at room temperature to remove cellular contaminants and debris. For sAA, samples are diluted 1:301 in diluted sample buffer, and read in a microplate reader (POLARstar Omega, BMG Labtech, Guelph, Canada) at 22 °C at a wavelength of 405 nm at 3 and 8 min after incubation at room temperature. For CORT, samples are incubated with enzyme conjugate solution at room temperature for 2 h, washed, incubated with enzyme substrate solution at room temperature for 30 min. After the addition of the “Stop Solution” to end the enzyme/substrate reaction, samples are read in a microplate reader (POLARstar Omega, BMG Labtech, Guelph, Canada) at 450 nm within 15 min. All samples are analyzed at least in duplicate.

Children are asked not to eat a major meal; consume food high in caffeine, sugar, or acidity; or brush their teeth for at least an hour before the session. In order to control for diurnal variation, each child’s sessions are scheduled at the same time of day. They are also asked to report the presence of any oral disease or injury, and whether or not they consumed any medications within the 12 h prior to the sessions. Adherence to these protocols is confirmed before beginning any sessions. If children have not followed the pre-testing protocols, enough time is passed to allow the protocol to be followed, or the child is rescheduled. Before the first collection of each visit, participants are instructed to swallow and rinse with water to clear the mouth of all saliva. Children are then not allowed to have anything to eat or drink throughout the rest of the session. Salivary samples for evaluation of sAA and CORT are collected throughout the intervention sessions. Specifically, samples are collected at baseline (after 10 min of baseline sitting), prior to (PRE), and immediately following experimental manipulation (i.e., exercise, TSST-C, rest), ERP/cognitive testing, and academic achievement testing (see Experimental Timeline).

### Blinding and randomization

Children are randomized after they complete the first visit. Each child receives each intervention in a counterbalanced, randomized fashion. The order of the intervention occurs through block randomization. Participants are unaware of their intervention order and are not informed of what intervention they will be completing during any given session until the actual intervention begins.

### Data monitoring

Access to individually identifiable private information will be restricted to the research coordinator, who will be the sole person responsible for maintaining that database. Identifiable and personal information will be kept separate from data, password-protected, and maintained in a secured room. All other individuals will be provided with only the unique record number assigned to each participant. All data will be protected via locked file cabinets or freezers behind locked doors, and password-protected personal computers located in Professor Hillman or Professor De Lisio’s laboratories. Anonymized data will be entered into REDCap (Research Electronic Data Capture). REDCap is a secure web application for building and managing surveys and databases. It allows a streamlined process for rapidly creating and organizing databases and allows for automated exporting of data to Excel and common statistical packages (SPSS, SAS, Stata, R) as well as a built-in project calendar, a scheduling module, ad hoc reporting tools, and other features. Data quality is promoted through verification of the data entered into REDCap using outcome specific range restrictions. REDCap also has a sophisticated logging module allowing for the tracking of all entered data. No protocol amendments are anticipated at this time; however, protocol amendments will be approved by the Northeastern University Institutional Review Board before modifications are made.

### Power and analytic strategy

A traditional power analysis was conducted to estimate the appropriate sample size necessary for detecting a mediating effect of sAA levels on the relationship between a single bout of aerobic exercise and acute changes in cognition given the inclusion of potentially confounding variables (e.g., age, sex, BMI, IQ, SES) based on a one-sided alpha of .01 to correct for multiple outcomes and a power of 0.8. For the total effect of exercise on the ERP and cognitive outcomes, an effect size was calculated from the results reported in Hillman et al. [9], which observed increased P3 amplitude, task performance, and scholastic achievement following acute exercise relative to rest in a sample of preadolescent children resulting in a moderate-to-large effect size (Cohen’s *d* = 0.79). Therefore, assuming a medium size of Cohen’s *f*2 = 0.15 for the total effect and 8 predictors (including exercise condition, subject ID, and covariates) in the regression model, power analysis indicate that a sample size of 82 participants will provide adequate power to detect the effects of a single bout of exercise on neuroelectric function and behavioral performance. For the mediation analysis, medium effect sizes [33] were assumed for both the direct effect of exercise on sAA levels (path a: coefficient = a) and the direct effect of sAA level on the outcomes controlling for treatment condition (path b: coefficient = b). Corresponding to Cohen’s [34] criteria for medium (13% of the variance) effect size, the values of a and b were set at 0.39. Applying the Sobel [35] first-order test, the indirect effect or the mediated effect of exercise on outcomes through sAA was indicated by the product of a and b and was tested. Empirical power results [36] showed that a sample size of *N* = 103 per study was required to detect the presence of mediation. Accordingly, we aim to recruit *N* = 120 to account for attrition.

We will investigate whether the effect of the experimental conditions (*x*) on brain function, cognition, or academic achievement (distal *y* outcomes) is mediated by sAA and CORT baseline levels (*i*) and sAA change (*s*) using a path modeling framework (Mplus v8) and conventional model-to-data fit indices [37]. Each model will be adjusted for the set of potentially confounding variables (e.g., age, sex, BMI, IQ, SES). We will interpret significant direct effects on each outcome as supporting our hypotheses. We aim to incorporate the change process of sAA (*s*) and CORT (*s*) within the same model (allowing s and s to covary). We will utilize the most current and recommended practices [38, 39] for testing moderated mediation framework that leverages the stability of the entire sample for model estimation (causal structure); 5000 bootstrap draws will be run to collect the bias-corrected bootstrapped confidence intervals (95% CI) and significant indirect effects will be decomposed in Mplus output (identical to the Sobel test [35]). If mediation is found, subsequent tests of moderation will be conducted. To test the effect of the treatment condition (i.e., exercise, TSST-C, rest) on univariate continuous outcomes (brain function, cognition, academic achievement), three separate models will be tested using planned comparisons with imposed group invariance constraints. Group comparisons in sAA mean can be simultaneously compared (Is sAA larger in exercise compared to TSST-C and/or rest?), while also comparing benefits in the continuous outcomes regressed on sAA.

### Dissemination plan

Upon completion of the study, we aim to disseminate and publicize the results to school administrators, policy makers, research study participants, and the general public by utilizing diverse strategies. Widespread dissemination will occur following study completion and publication, with the main results as well as secondary and ancillary results by employing the following techniques: (a) media coverage through press releases and interviews targeted to local and national news outlets; (b) production of a research summary document and “facts sheet” targeted to the general public, which summarizes key study conclusions; (c) production of flyers, posters, brochures, and research briefs targeted to broad audiences; (d) study newsletters targeted to study participants; (e) distribution of dissemination materials to community agencies, professional societies and health-related websites and list-serves; and (f) mailing personal thank you letters that include the main findings to research study participants.

## Discussion

The SNEACY study is a large, ongoing randomized crossover trial with a goal of investigating phasic changes in SNS activation (indicated by differential sAA and CORT trajectories) as a potential mechanism for the transient beneficial effect of moderate intensity exercise on brain function, cognition, and academic achievement in cognitively normal children. We predict that following the cessation of moderate intensity exercise performance on executive function tasks will improve, with enhanced neuroelectric functioning and that these improvements will be linked to changes in sAA. In addition, both sAA and cortisol are expected to increase in response to both exercise and the TSST-C in children. This is a well-designed and controlled study, with the exercise prescription individualized to each child reflecting their specific moderate intensity exercise. We further predict that academic performance will be greatest following exercise.

While this is a well-designed, detailed study, it is not without its limitations. The findings from this study will not allow us to determine whether different doses of exercise (i.e., duration, intensity, etc.) result in differences is neuroelectric profiles and cognition. However, we do expect to observe differences in performance based on an individual’s fitness and body composition. Our active control intervention is designed to increase psychosocial stress for the participants, and to increase their heart rate as a result. However, our passive control group is seated at quiet rest and prior research does not indicate cognitive benefits following seated rest [8–10]. In this sense, this is a well-controlled study with the inclusion of positive (TSST-C) and negative (seated rest) conditions. Finally, it would be impossible to measure all individual differences across our participants, which could influence their performance following interventions. There are several challenges that large-scale studies such as this one face. Primarily, ensuring accurate saliva measures from children may be a challenge, and relies on day of pre-intervention compliance from children and their parents. In addition, children must produce enough saliva in a set amount of time. Maintaining compliance and interest over a number of visits for children may be challenging as well.

Taken together, the SNEACY study will be among the first to investigate the molecular underpinnings of the acute exercise-induced changes in cognition and brain in children through a combination of molecular, neuroimaging, behavioral, and academic achievement measures. Previous data indicates that a single bout of aerobic exercise increases sAA [15, 40, 41] and salivary CORT [42–45], reflecting increased activation of the SNS and glucocorticoid system following an exercise bout, respectively. Acute exercise has also been related to enhanced cognition [20] and acute elevations in sAA and CORT [42, 46]. For example, in a study with older adults, memory was enhanced after a brief exercise bout, and changes in sAA following exercise correlated with improvements in memory [20]. However, data from the present study will aid in our understanding of the relationship between exercise, cognition, and the SNS and adrenal systems.

Therefore, we will examine changes in behavioral and brain measures following a single, brief bout of exercise, and seek to explain these shifts through phasic changes in sAA that are reflective of SNS activity. Through the collection of salivary, neuroimaging, and behavioral outcomes in the proposed randomized crossover experiment, we can begin to establish the multidimensional nature of the transient effects of a single bout of exercise on the underlying molecular and neural changes that promote cognitive health and academic achievement during preadolescent childhood.

## Data Availability

Not applicable. Data collection is ongoing. When complete, the datasets will be available from the corresponding author on reasonable request.

## Abbreviations

ADHD: Attention deficit hyperactivity disorder
BMI: Body mass index
CRF: Cardiorespiratory fitness
CORT: Cortisol
DXA: Dual-energy X-ray absorptiometry
EEG: Electroencephalogram
ERPs: Event-related brain potentials
HR: Heart rate
IEP: Individualized education plan
IQ: Intelligence quotient
ISI: Inter-stimulus interval
MVPA: Moderate-to-vigorous physical activity
PA: Physical activity
sAA: Salivary alpha-amylase
SNEACY: Sympathetic Nervous System & Exercise Affects Cognition in Youth
SES: Socio-economic status
SNS: Sympathetic nervous system
TSST: Trier Social Stress Test
RPE: Ratings of perceived exertion
